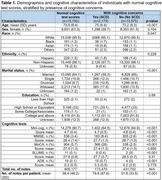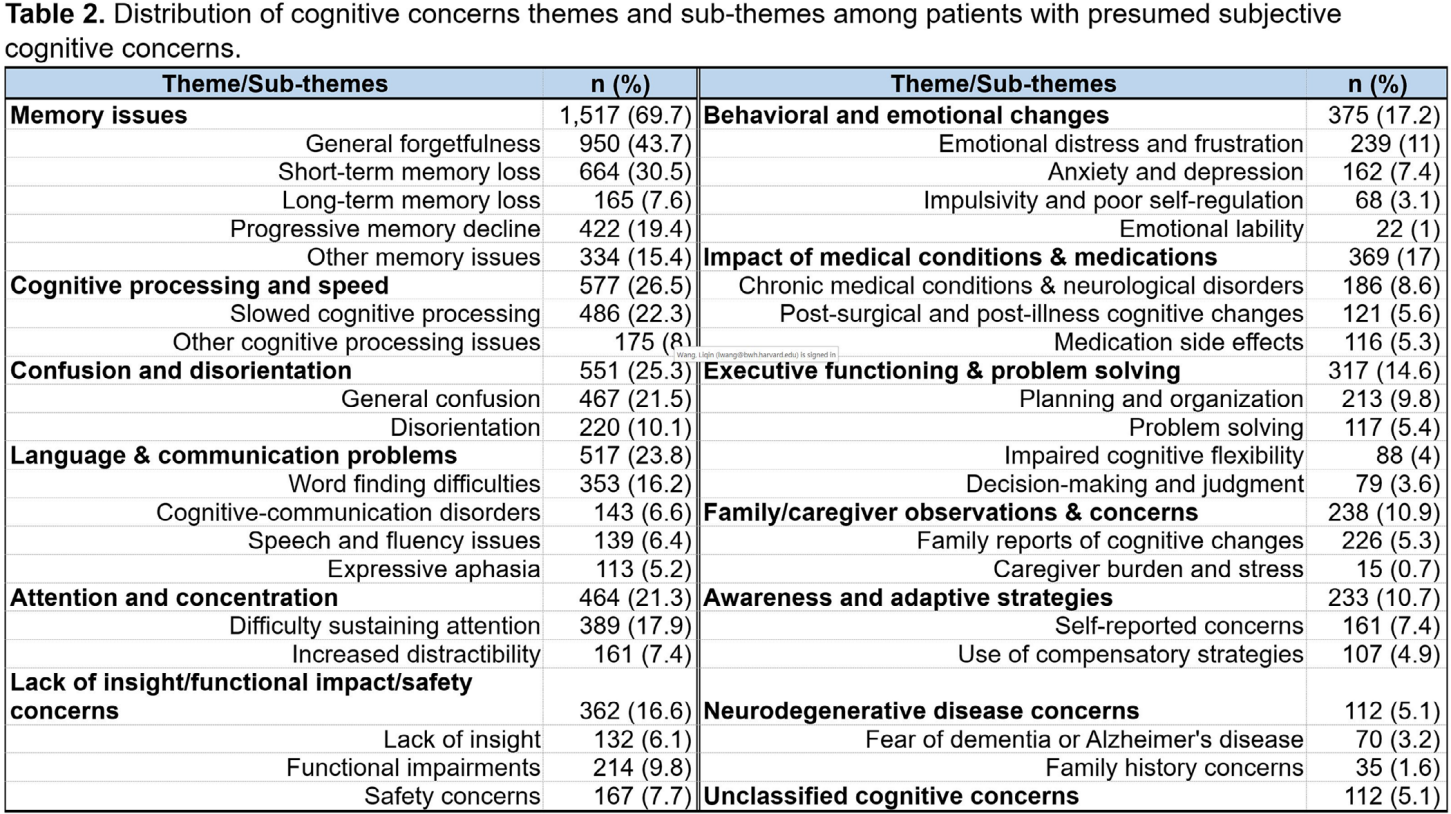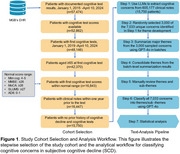# Classifying Cognitive Concerns in Subjective Cognitive Decline: Insights from Clinical Notes

**DOI:** 10.1002/alz70857_107715

**Published:** 2025-12-26

**Authors:** Liqin Wang, Rebecca E. Amariglio, Li Zhou, Gad A. Marshall

**Affiliations:** ^1^ Brigham and Women's Hospital, Harvard Medical School, Boston, MA, USA; ^2^ Center for Alzheimer's Research and Treatment, Department of Neurology, Brigham and Women's Hospital, Harvard Medical School, Boston, MA, USA; ^3^ Department of Neurology, Massachusetts General Hospital/Brigham & Women's Hospital, Boston, MA, USA; ^4^ Center for Alzheimer Research and Treatment, Department of Neurology, Brigham and Women's Hospital, Boston, MA, USA

## Abstract

**Background:**

Subjective cognitive decline (SCD) referring to self‐ or informant‐reported memory or cognitive concerns despite normal test performance, offers a critical window for early intervention. However, because SCD lacks a standardized diagnostic code and cognitive concerns are typically documented only in free‐text electronic health records (EHR) notes, it often goes unrecognized by traditional data queries. There is limited research on the characteristics of patients with SCD and the types of cognitive concerns documented during this early stage.

**Methods:**

This retrospective cohort study analyzed EHR data from Mass General Brigham (MGB) for patients ≥65 years who underwent initial cognitive assessments between January 1, 2019, and April 10, 2024. For patients with normal cognitive test scores, clinical notes from the year prior were extracted. A two‐tiered large language model (LLM) approach identified cognitive concerns: Med42‐v2‐8B processed large volumes of notes to flag potential cognitive concerns (high sensitivity); GPT‐4o (via Microsoft Azure) refined results to improve precision. GPT‐4o also summarized themes and sub‐themes from a random subset, which were then applied to all concerns. Statistical analyses compared patients with and without documented concerns and identified the most common themes and subthemes and their distribution.

**Results:**

Our study included 15,750 patients with normal cognitive test scores, of which 9,651 (61.3%) were female, with a mean age of 73.5 (SD 6.4) years. A total of 605,177 clinical notes were analyzed, identifying cognitive concerns or SCD in 2,175 patients (13.8%). LLMs identified 12 major themes and 34 sub‐themes, leveraging 42.7% (*n* = 3,000 out of 7,033 unique concerns). Among patients with cognitive concerns, the most frequent themes were memory issues (69.7%), followed by cognitive processing and speed (26.5%), confusion and disorientation (25.3%), and language and communication problems (23.8%). Additional themes included lack of insight/functional impact/safety concerns (16.6%), behavioral and emotional changes (17.2%), impact of medical conditions/medications (17%), executive function and problem solving (14.6%), and others.

**Conclusion:**

The study demonstrates the feasibility of using LLMs to extract and classify cognitive concerns in patients with presumed SCD. The identified themes provide valuable insights into common cognitive issues. Future research should examine how these symptoms in related to dementia progression.